# The Intricacy of Setting Up a Service to Bridge the Gap in Skin Malignancy Treatment for the Frail Population

**DOI:** 10.7759/cureus.86853

**Published:** 2025-06-27

**Authors:** Aude Perusseau-Lambert, Charlotte B Miller

**Affiliations:** 1 St Andrew's Centre for Plastic Surgery and Burns, Broomfield Hospital, Mid and South Essex National Health Service (NHS) Foundation Trust, Chelmsford, GBR

**Keywords:** cutaneous malignancy, electrochemotherapy, frail patients, non-surgical cancer management, patient-centered care multidisciplinary approach, service development, treatment gap

## Abstract

Skin malignancies such as basal cell carcinoma, squamous cell carcinoma, and melanoma are increasingly prevalent in the UK, particularly among frail and elderly patients who are often unsuitable for surgical intervention due to comorbidities, anticoagulation, or impaired wound healing. This study describes the development and implementation of an outpatient electrochemotherapy (ECT) service designed to address this treatment gap. ECT combines local chemotherapy with electroporation to enhance drug uptake by tumour cells and is a minimally invasive, National Institute for Health and Care Excellence (NICE)-approved technique that can be performed under local anaesthesia.

A pilot study conducted between February 2022 and July 2024 demonstrated the clinical and economic benefits of this approach, with patients experiencing fewer complications, reduced wound care requirements, and faster recovery compared to conventional surgical excision. The service was established through close collaboration between plastic surgeons, oncologists, pharmacists, nurses, and administrative teams. Key components included the creation of streamlined workflows, simplified consent documentation, tailored patient information, and a structured care pathway. Challenges encountered during implementation, such as interdepartmental communication, pain management, and wound care education, were addressed through ongoing quality improvement efforts, including regular multidisciplinary meetings, clinical audits, and outcome tracking (patient-reported outcome measures).

Electrochemotherapy offers a safe, effective, and accessible alternative for the treatment of skin malignancies in frail patients. Despite the logistical and organisational challenges involved in its implementation, the service has shown clear benefits in terms of reduced morbidity, improved recovery, and patient satisfaction, supporting its potential for broader adoption across the National Health Service (NHS).

## Editorial

Identify the need for a new clinical service: introduction to skin malignancies and the frail population

Skin malignancies, particularly basal cell and squamous cell carcinomas, as well as melanomas, are a significant public health concern in the United Kingdom, where approximately one in four men and one in five women will develop some form of skin cancer during their lifetime [1]. The incidence of melanoma has escalated sharply over recent decades, with rates nearly tripling among men and more than doubling among women [2]. Melanoma is now the fourth most prevalent cancer among men and the fifth among women. Despite advancements in skin cancer treatment that have improved patient outcomes, frail and elderly individuals encounter a distinct set of challenges. Many of these patients are deemed unsuitable for conventional surgical interventions due to comorbidities associated with extensive disease, compromised wound healing, and ongoing anticoagulation therapy [3,4]. In line with the “NHS long-term plan” and development of innovation in healthcare [5], this essay delves into the complexities of establishing a new day-case electrochemotherapy (ECT) service, meticulously designed to bridge the treatment gap for the vulnerable patient demographic. 

Challenges of treating skin malignancies in frail patients 

The consequences of untreated skin malignancies can be dire, with considerable morbidity. Extensive tissue damage and disfigurement with potential for local invasion into surrounding structures (perineural, perivascular, and bony tissues) can occur. As well as the development of metastatic disease to critical organs, including the brain, liver, and lungs. These advanced malignancies can severely impair the patients’ quality of life and result in mortality. Historically, surgical intervention has been the cornerstone of treatment for skin malignancies. However, frail patients often face prohibitive risks associated with surgery, including impaired wound healing, elevated bleeding risks, or extensive disease necessitating intricate reconstructions. The combination of these conditions and the requirement for a general anesthetic can be too risky, rendering these patients ineligible for surgery. Consequently, there is a pressing need for alternative, minimally invasive treatments that balance efficacy and safety. 

Introducing the electrochemotherapy service 

In response to this pressing need, we proposed the establishment of an electrochemotherapy service within the plastic surgery outpatient department of our hospital. The patients were referred to the Plastic Surgery and Laser Consultant in charge of this project by the other Plastic Surgery Consultants of the department when deemed appropriate, and surgery was not suitable. Electrochemotherapy is National Institute for Health and Care Excellence (NICE)-approved and represents an innovative treatment modality that integrates targeted intra-tumoral injection of a chemotherapy agent combined with brief, high-voltage, electrical pulses to enhance drug uptake by cancer cells [6,7]. These pulses temporarily permeabilise the tumour cell membranes, facilitating more effective drug absorption and a potent cytotoxic effect. By concentrating the treatment directly on the tumour site, electrochemotherapy minimises collateral damage to surrounding healthy tissues and mitigates systemic side effects. This approach is performed under local anaesthesia, making it an optimal choice for patients unsuitable for traditional surgical or general anesthesia. 

To address skin cancer, the options available were: (1) “do nothing”, with a risk of progression of the skin malignancy and invasion to deeper tissue and anatomical structures, (2) cryotherapy or topical immune response modifiers: suitable for for superficial skin malignancies with a risk of creating an open wound, (3) oncology (radio/immunotherapy): systemic side effects, (4) surgery: in this case, the selected patients were not suitable for surgical intervention due to comorbidities such as impaired healing or extended lesions, (5) electrochemotherapy: targets superficial and invasive skin malignancy, minimal/no wound. This is a palliative management option.

Procedure workflow on treatment days 

Electrochemotherapy was performed in a minor-operation room within the outpatient setting. Patients and their lesions were assessed prior to obtaining informed consent. In accordance with European Standard Operating Procedures in Electrochemotherapy (ESOPE) guidelines, up to seven lesions measuring less than 3 cm in diameter could be treated per session using a total of 5000 IU of bleomycin. Larger lesions (>3 cm) were typically treated individually and might require additional sessions.

Clinical photographs were taken pre-treatment to document the appearance of the lesion for the medical record and repeated to facilitate the assessment of treatment response during follow-up consultations. To minimise discomfort, topical anaesthetic cream was applied to the lesions for at least 20 minutes to ensure adequate superficial analgesia. Intralesional local anaesthetic (a combination of lidocaine 1% and bupivacaine 0.25%) was subsequently administered for deeper and prolonged analgesia.

The total dose of bleomycin was distributed among the lesions and injected intratumorally. Electroporation was then performed using a needle-based probe applied directly to the solid tumours. The length of the needle (5 mm, 10 mm, or 20 mm) was selected according to skin and lesion thickness. Electroporation parameters followed the manufacturer's standards: a constant voltage of 1300 V, pulse frequency of 250 kHz, and variable current (1-30 A) determined by tissue conductivity. This process transiently permeabilised tumour cell membranes, enhancing intracellular uptake of bleomycin. Electroporation increases drug absorption by up to 1000-fold through passive diffusion while the cell membranes remain permeable [8]. A major advantage of this procedure is the minimal tissue disruption and absence of bleeding. Needle entry points typically close within one hour [8]. Post-procedure, a silver-based dressing was applied, and patients were reviewed in the dressing clinic one week later.

**Figure 1:**
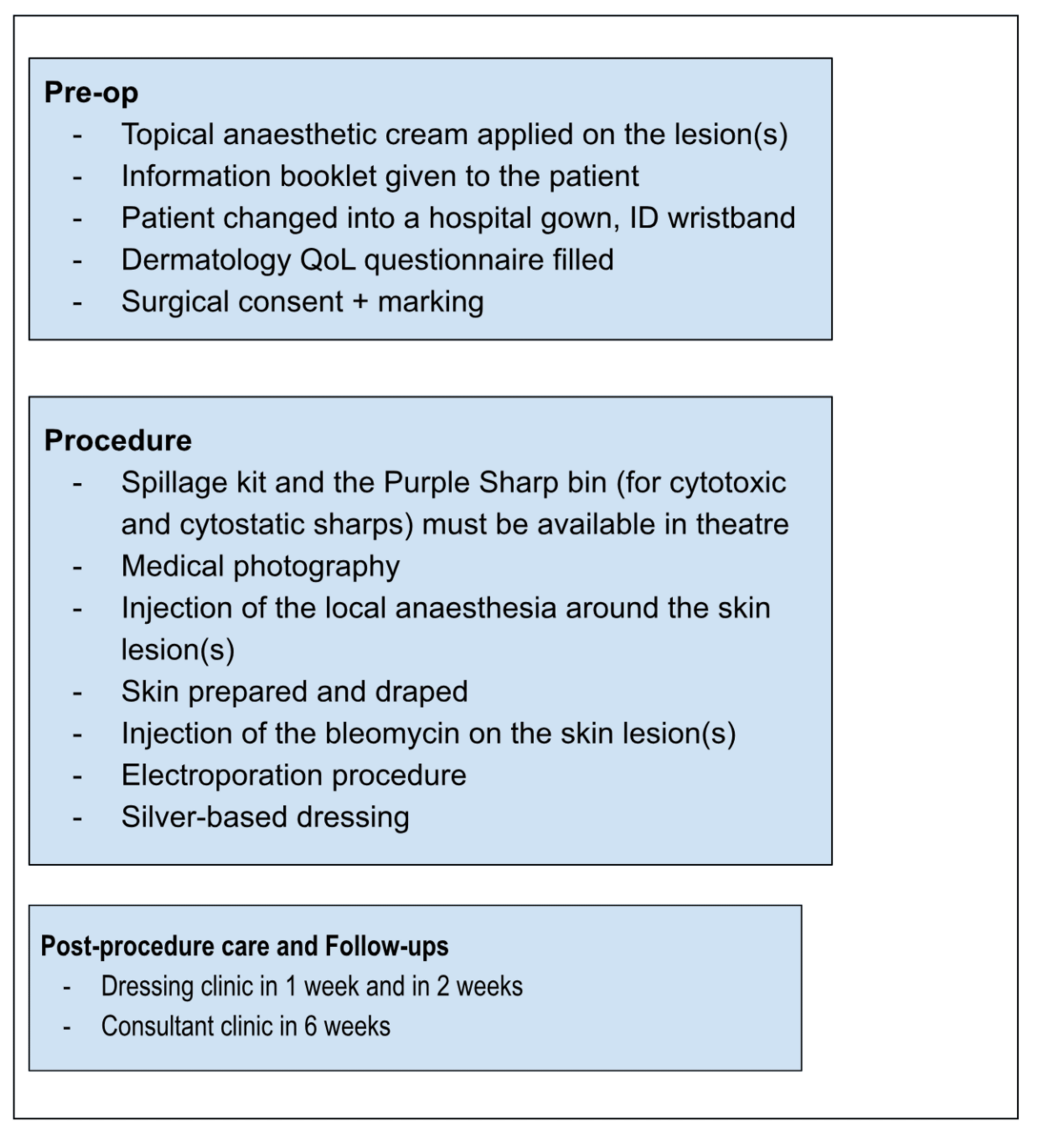
Patient care flow on the day of the procedure

At each follow-up visit (one week, two weeks, and six weeks post-procedure), the treated areas were clinically assessed and further photographs obtained to monitor progress. At the six-week review, any persistent lesions were evaluated for repeat electrochemotherapy. A care pathway was established to support each team member in delivering coordinated patient care on treatment days, ensuring an efficient process and streamlined workflow (Figure 1).

Benefits of local electrochemotherapy for frail patients 

Electrochemotherapy, through electroporation, performed under local anaesthesia, offers an exceptionally effective precision delivery of non-thermal ablation and a minimally invasive treatment option for skin malignancies in frail patients. The procedure has demonstrated fewer side effects, shorter recovery periods, and superior patient outcomes compared to traditional surgical interventions. From a cost-efficiency perspective, electrochemotherapy reduces the need for extensive postoperative care and follow-up appointments, thereby lowering the overall treatment costs for both patients and healthcare providers. The service also enhances accessibility by being available as a day-case procedure under local anaesthesia, which does not necessitate discontinuation of anticoagulant medication, making it a viable option for patients who may be unable to tolerate general anaesthesia or prolonged hospital stays.

Pilot study on electrochemotherapy in frail patients 

Between February 2022 and July 2024, we conducted a pilot study evaluating the efficacy and cost-effectiveness of electrochemotherapy for the treatment of frail patients with cutaneous malignancies. The findings demonstrated that patients undergoing electrochemotherapy experienced no postoperative complications beyond mild erythema, minimal and transient skin breach or ulceration, and benefited from accelerated recovery and significantly reduced wound care requirements. In our experience, the need for post-treatment wound care (dressing clinic) was halved compared to a matched cohort undergoing conventional surgical excision and reconstruction.

One illustrative case involved a patient who had previously required 18 months of wound care following excision and split-thickness skin grafting for a squamous cell carcinoma on a severely oedematous leg. In contrast, the same patient was later treated with electrochemotherapy for a comparable lesion on the contralateral leg, requiring only two nurse-led outpatient dressing appointments, and an overall improved cosmetic outcome as there was no contour deformity on their leg (vs the deformity left following excision and split thickness skin graft). This striking comparison highlights electrochemotherapy’s potential to improve clinical outcomes while reducing both patient burden and demands on healthcare resources.

Another noteworthy case involved a patient with multiple superficial skin lesions affecting nearly half of the face, including a recurrent basal cell carcinoma on the forehead and squamous cell carcinomas on both temples and cheeks. Given the extent of involvement, surgical intervention was deemed unsuitable. Electrochemotherapy was administered, and the lesions showed complete regression following a single treatment session.

Challenges in setting up the electrochemotherapy service 

Establishing a new service in a hospital setting for frail patients can present considerable challenges. Coordinating diverse departments and professionals, including plastic surgeons, oncology consultants, pharmacists, and outpatient staff, proved to be a complex task. Each team had its own terminologies and priorities, necessitating careful adjustments in communication to ensure cohesive collaboration. For instance, simplifying clinical jargon for nursing staff while conveying logistical details to pharmacists required meticulous communication management. 

Equally challenging was the management of patient logistics. Patients were required to undergo blood tests (including full blood count, urea and electrolytes, International Normalized Ratio (INR)) three to five days prior to the procedure, necessitating precise coordination with the patient, their next of kin, and their primary care providers. The patients and their next of kin or carers required detailed explanations about the electrochemotherapy procedure, postoperative care, and follow-up schedules, often involving additional time to ensure full comprehension. Communicating these details in clear, non-technical language, while also addressing the concerns of their relatives, added another layer of complexity. This level of coordination was crucial to ensure a seamless procedure day, minimise patient anxiety, and achieve optimal clinical outcomes. 

Ongoing evaluation and quality improvement 

The success of the electrochemotherapy service depends on continuous evaluation and adaptation to maintain high standards of care. Regular multidisciplinary meetings with surgeons, oncologists, pharmacists, and nurses facilitate discussions on patient outcomes, complications, and service improvements. Logistical challenges in coordinating pre-procedure assessments led to a streamlined pathway, reducing delays and improving efficiency. Strict patient safety protocols ensure adherence to national guidelines, with structured consent processes and regular audits assessing complications and service efficiency. An audit identifying gaps in post-procedure pain management led to standardized analgesia protocols (application of topical anaesthetic cream), enhancing patient comfort. 

Systematic data collection, including monitoring tumour response rates (using the “World Health Organization criteria for tumour response assessment” ref) and patient-reported outcomes (Dermatology Life Quality Index), informs service improvements. Early reviews highlighted the need for better wound care education, prompting structured outpatient follow-ups to support healing and reduce unnecessary clinic visits. Quality improvement efforts refine workflows, enhance communication, and integrate new technologies. Recognizing transportation difficulties for frail patients led to a nurse-led community dressing service, reducing hospital visits while maintaining continuity of care. 

Ongoing staff training ensures best practices, with team participation in workshops and research. Regular communication between the staff is also needed to ensure any areas of concern can be addressed. We designed “care-flow” to support staff involvement with the patient on the day, and what was required from them (Figure 1). Patient education is key to treatment adherence and satisfaction. Clear pre- and post-treatment guidance reduces anxiety and improves compliance, leading to the introduction of a simplified patient information leaflet based on feedback. Involving the next of kin was key to ensuring patient care and compliance with follow-up. 

Compliance with GDPR principles ensures lawful, fair, and secure data handling. Patients are informed of data use, with only essential information collected and securely stored. A digital patient record system improved security while streamlining access for authorised professionals. 

Integration into broader healthcare systems 

The integration of the electrochemotherapy service into the broader healthcare system involves collaboration with various stakeholders, including the patients, the plastic surgery team, the primary care providers, oncologists, the outpatient administrating team, and community health services. Establishing strong relationships with these stakeholders ensures comprehensive care throughout the patient’s treatment journey. Coordination with primary care providers is crucial for managing patients' overall health and addressing comorbid conditions that may impact their treatment. 

Community health services play a supportive role, particularly for patients requiring assistance with transportation, medication management, or follow-up care. Engaging with these services helps bridge gaps in care and offers a more holistic approach to patient management. Additionally, aligning the electrochemotherapy service with national or regional cancer care frameworks can enhance its accessibility and sustainability. Collaboration with cancer networks and organisations helps integrate the service into broader cancer care strategies, ensuring it effectively meets the needs of the patient population. 

Future directions 

Looking forward, several potential developments could further enhance the electrochemotherapy service and expand its impact. Research into optimising electrochemotherapy protocols, such as exploring different chemotherapy agents or electrical parameters, could improve treatment efficacy and patient outcomes. Additionally, investigating the use of electrochemotherapy in combination with other therapeutic modalities, such as immunotherapy or targeted therapies, may present new opportunities for addressing complex cases. 

Expanding the service to include other types of skin malignancies or even non-skin cancers (e.g., keloid scar) could broaden its applicability and benefit a wider patient population [9]. Furthermore, ongoing research into patient selection criteria and treatment planning could refine the indications for electrochemotherapy, ensuring its use in the most suitable cases. Developing predictive models to identify patients likely to benefit most from electrochemotherapy could further enhance treatment precision and improve overall outcomes. All those avenues are aimed to be answered by the involvement of the unit providing this treatment, with a recognised shared national/international database. 

Conclusion 

The implementation of an electrochemotherapy service represents a meaningful advancement in the management of skin malignancies, particularly for frail and elderly patients who present specific challenges with conventional surgical approaches. By offering a minimally invasive and effective treatment that can be delivered under local anaesthesia, electrochemotherapy addresses a critical gap in oncological care and contributes to improved quality of life for this vulnerable population.

Despite the challenges involved in setting up and coordinating the service, the demonstrated benefits, including reduced postoperative complications, expedited recovery, cost-effectiveness, and improved accessibility, underscore its value within the healthcare landscape. Following the successful pilot, a business case was developed and is currently under review by the finance team and departmental managers for further approval.

Ongoing evaluation, quality improvement initiatives, and integration into wider healthcare systems are essential for sustaining and enhancing the service’s impact. As research and development continue to refine electrochemotherapy techniques and identify new applications, the service is well-positioned to make a meaningful contribution to bridging the treatment gap for frail patients with skin cancer.
